# Supplementary material for: Computational design of small transcription activating RNAs for versatile and dynamic gene regulation
Source: Nat Commun. 2017 Oct 19;8:1051. doi: 10.1038/s41467-017-01082-6 (PMC5648800; doi:10.1038/s41467-017-01082-6)
Supplement: Supplementary file 1 — Supplementary Information [file 41467_2017_1082_MOESM1_ESM.pdf]

Computational design of Small Transcription Activating RNAs (STARs) for versatile and dynamic gene regulation

Supplementary Table 1. Sequence of promoter and RBS variants. Promoter 1 (BBa_J23119_SpeI) is a variant of BBa_J23119 obtained from the iGEM Registry of Standard Biological Parts (parts.igem.org). Promoters 2-5 were derived from a previously published promoter library[1]. RBS variants were generated by inverse PCR using degenerate oligonucleotides and five variants chosen that demonstrated distinct expression strengths of sfGFP from target variant 5 in the presence of cognate STAR. The stability hairpin used for the no target RNA control in **Supplementary Fig. 12**. was derived from a previously published library[2].

Name	Sequence (5' to 3')
Promoter 1	TTGACAGCTAGCTCAGTCCTAGGTATAATACTAGT
Promoter 2	AAAAAGAGTATTGACTTCGCATCTTTTTGTACCTATAATGTGTGG
Promoter 3	TTGACAATTAATCATCCGGCTCGTAATTTATGTGG
Promoter 4	AAAAAATTTATTTCGCATCTTTTTGTACCTATAATGTGTGG
Promoter 5	TTGACAATTAATCATCCGGCTCGTAGGGTTTGTGG
RBS 1	AGGAGGAA
RBS 2	GTAACGGA
RBS 3	GTATTGGA
RBS 4	TATTGGGA
RBS 5	TAGAGGTG
Stability hairpin	ACGTCGACTCTCGAGTGAGATTGTTGACGGTACCGTATTTT

Supplementary Table 2: Sequences of toehold switch and trigger used. The best performing toehold switch and trigger (referred to as forward-engineered 1) were derived from the original paper by Green *et al.*[3]

Name	Sequence (5' to 3')
Toehold Switch 1:<br>Switch 1 - linker	GGGTCTTATCTTATCTATCTCGTTTATCCCT<br>GCATACAGAAACAGAGGAGATATGCAATGATAAACGAG<br>AACCTGGCGGCAGCGCAAAAG
Toehold Trigger 1:<br>Stability Hairpin -Trigger 1	GGGACTGACTATTCTGTGCAATAGTCAGTAAA<br>GCAGGGATAAACGAGATAGATAAGATAAGATAG

Supplementary Table 3. Strains used in this study. Strains containing genomic insertions were created using the clonetegration platform[4] to integrate the inserts using the HK022 plasmid into the *attB* site of the *E. coli* genome. Successful integrations were identified by antibiotic selection and colony PCR according to the published protocol[4].

Strain	Strain Genotype	Genomic Insertion
E. coli TG1	K-12 *supE thi-1 Δ(lac-proAB) Δ(mcrB-hsdSM)5, ($r_K^-m_K^-$)* F' [*traD36 proAB*$^+$ *lacI*^{q} *lacZΔM15]*	N/A
E. coli TG1<br>target variant 5<br>*sfgfp*	K-12 *supE thi-1 Δ(lac-proAB) Δ(mcrB-hsdSM)5, ($r_K^-m_K^-$)* F' [*traD36 proAB*$^+$ *lacI*^{q} *lacZΔM15]*<br>*attB::target variant 5 sfgfp CmR*	*attB::target variant 5 sfgfp(* Promoter 1 – Target 5 – RBS 1 – sfGFP – TrrnB – CmR)
E. coli BW25113	F$^-$, *Δ* (araD-araB)567, lacZ4787(del)::rrnB-3, LAM$^-$, rph-1, *Δ* (rhaD-rhaB)568, hsdR514	N/A
E. coli BW25113<br>Δ*cheZ*	F$^-$, *Δ* (araD-araB)567, lacZ4787(del)::rrnB-3, LAM$^-$, rph-1, *Δ* (rhaD-rhaB)568, hsdR514, *ΔcheZ734::kan*	N/A
E. coli BW25113<br>Δ*cheZ*<br>target variant 5<br>*cheZ*	F$^-$, *Δ* (araD-araB)567, lacZ4787(del)::rrnB-3, LAM$^-$, rph-1, *Δ* (rhaD-rhaB)568, hsdR514, *ΔcheZ734::kan, attB::target variant 5 cheZ CmR*	*attB::target variant 5 cheZ* (Promoter 1 – Target 5 – RBS 1 – CheZ – TrrnB – CmR)
E. coli DH5 alpha pir	F-, *Δ(argF-lac)169*, *ϕ*80d*lacZ58*(M15), *ΔphoA8*, *glnX44*(AS), *λ*$^-$, *deoR481*, *rfbC1*, *gyrA96*(NalR), *recA1*, *endA1*, *thiE1*, *hsdR17, Δ uidA3::pir*	N/A

Supplementary Table 4: Primers used for reverse transcription and quantitative PCR

Primer Name	Sequence (5' to 3')
RT.sfGFP	TTATTTGTAGAGCTCATCCATG
sfGFP.Fwd	CACTGGAGTTGTCCCAATTCT
sfGFP.Rev	TCCGTTTGTAGCATCACCTTC

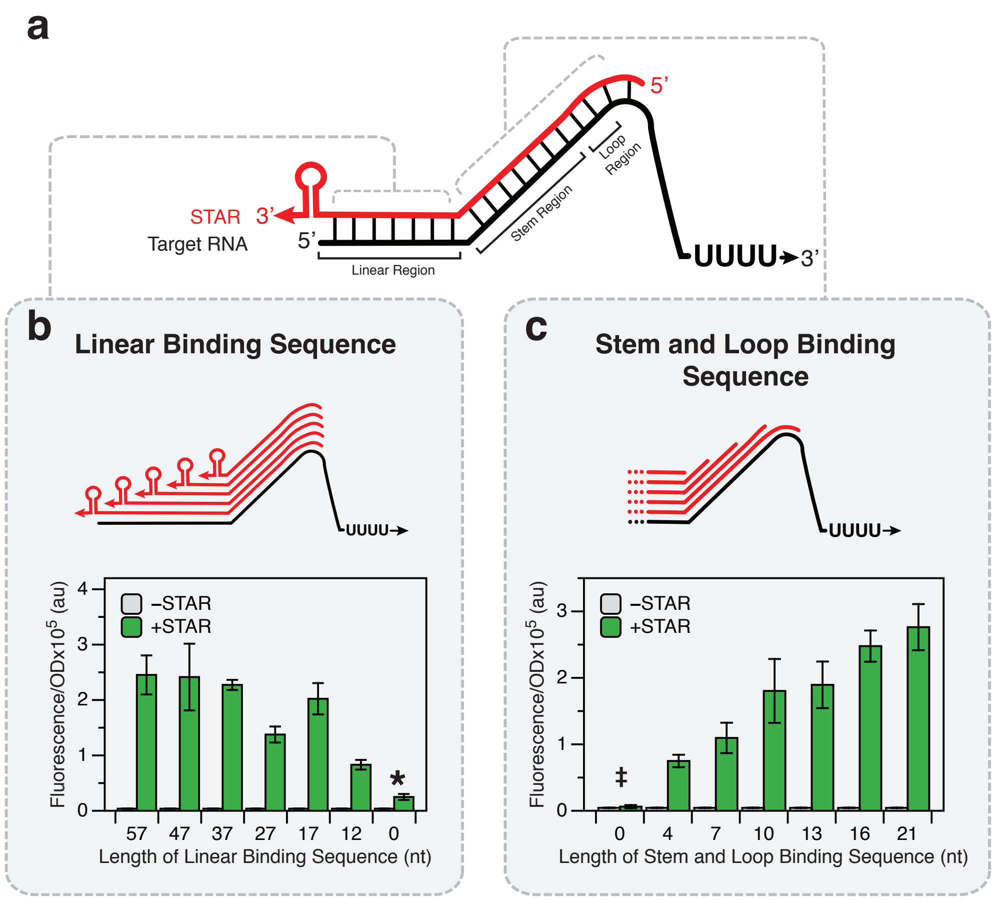

Supplementary Figure 1. Determining a STAR design motif. (**a**) Schematic of a STAR and target RNA complex with the different interaction regions annotated. Schematics and fluorescence characterization of STAR variants that were used to determine optimal lengths of (**b**) the linear binding sequence and (**c**) the stem and loop binding sequence of the AD1 STAR[5]. STAR variants were created by truncating either the (**b**) 3' or (**c**) 5' end of the STAR while the target RNA was kept constant. Characterization revealed the optimal lengths were 21 nucleotides (nt) for the stem and loop binding sequence and ~40 nt for the linear binding sequence. In addition, it was observed that neither the stem and loop binding sequence itself (0 nt in (**b**) indicated by *) or the linear binding sequence itself (0 nt in (**c**) indicated by ‡) of the STAR were sufficient to appreciably activate transcription. This reveals a design motif to create orthogonal and functionally diverse variants by varying the linear region of target:STARs while maintaining a constant stem and loop region. Fluorescence characterization (measured in units of fluorescence/optical density [OD] at 600 nm) was performed on *E. coli* cells transformed with the AD1 target DNA plasmid in the absence (-STAR) and presence (+STAR) of a DNA plasmid encoding a cognate STAR length variant. Data represent mean values and error bars represent s.d. of n = 9 biological replicates.

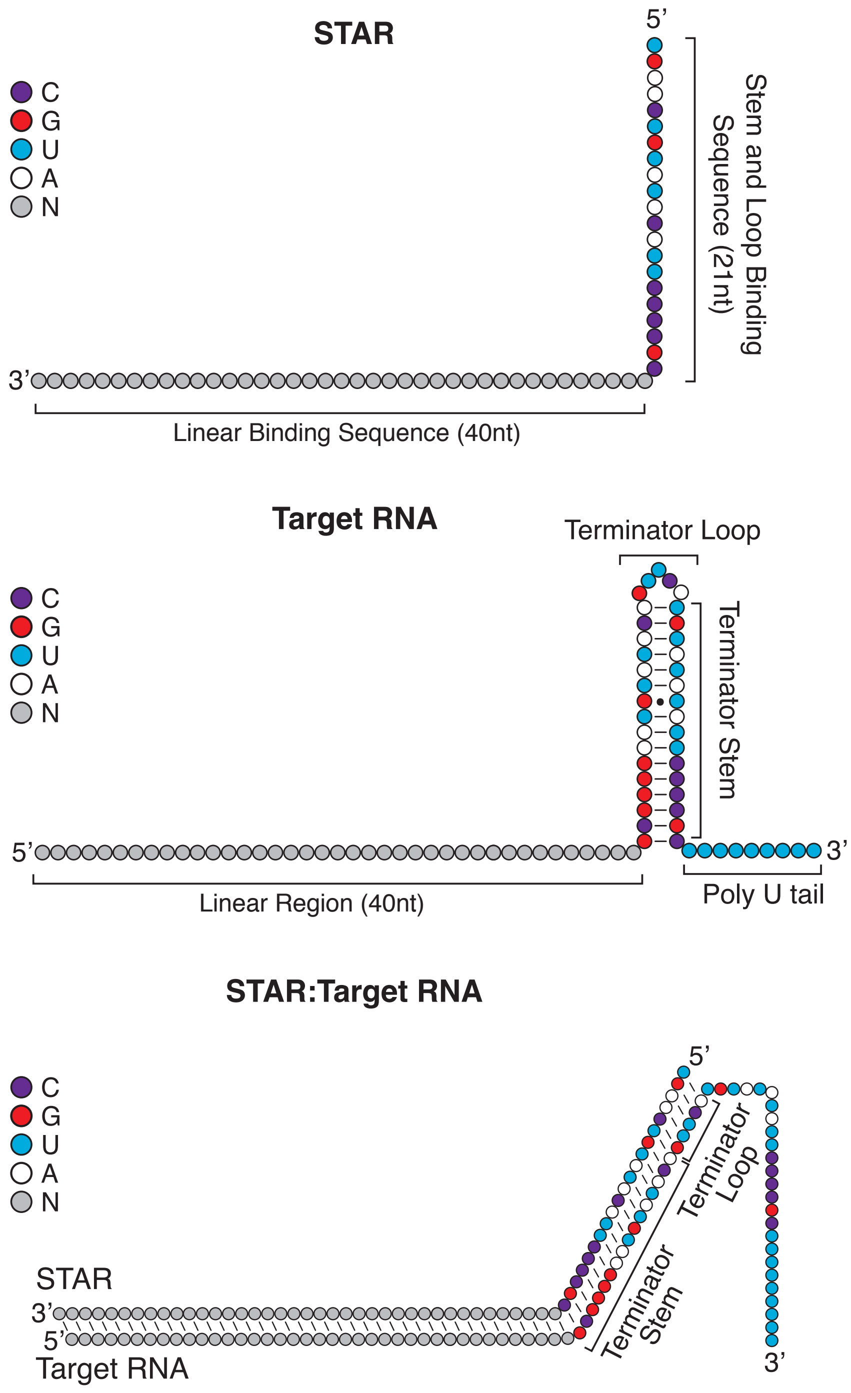

Supplementary Figure 2. Schematic of the STAR design motif used in NUPACK. Schematic of the sequence and structure constraints for the STAR, target RNA and STAR-target RNA complex used in the NUPACK design algorithm[6, 7]. Nucleotides colored according to identity with N representing an unconstrained nucleotide that is designed by NUPACK. See **Supplementary Note 1** for a description of the NUPACK script used.

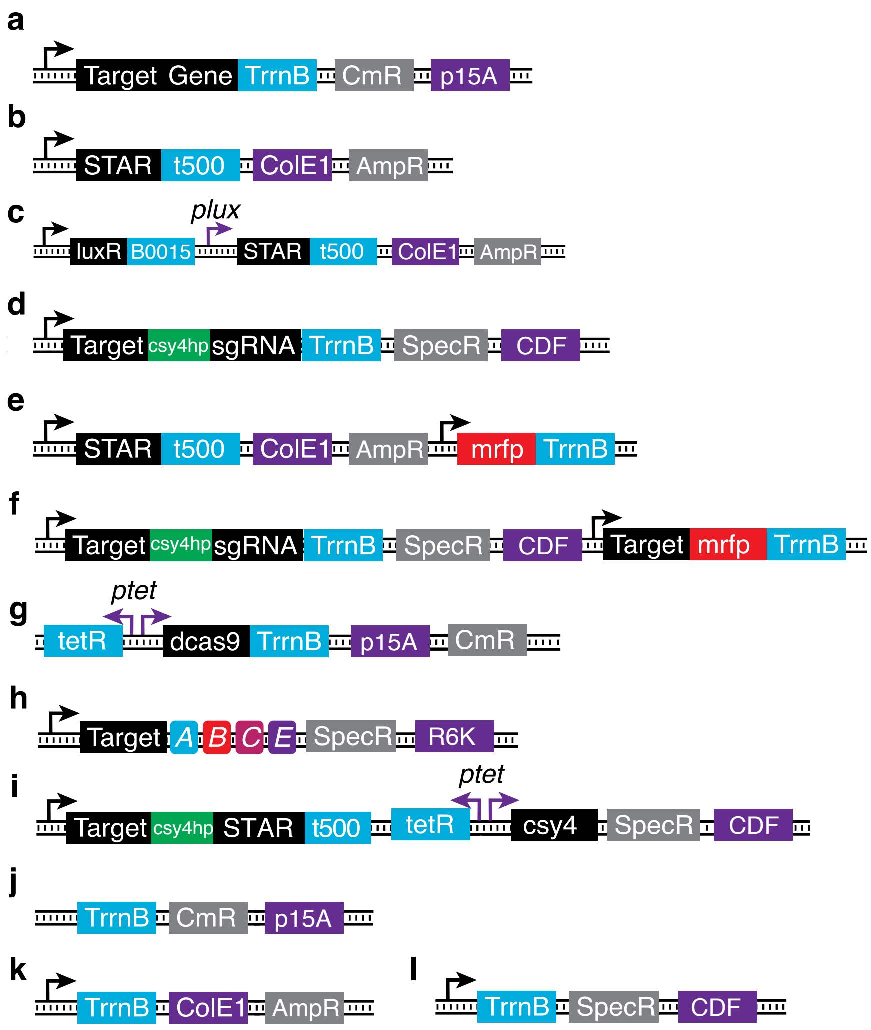

Supplementary Figure 3. Schematic of representative DNA plasmid maps used in this study. (**a**) Target RNA expressing plasmid, (**b**) STAR expressing plasmid, (**c**) AHL inducible STAR expressing plasmid, (**d**) STAR regulated sgRNA plasmid, (**e**) STAR and mRFP expressing plasmid, (**f**) STAR regulated sgRNA and mRFP plasmid, (**g**) dCas9 expressing plasmid, (**h**) STAR regulated deoxyviolacein plasmid, (**i**) Stage 2 activation-activation cascade expressing plasmid, (**j**) no target RNA control plasmid, (**k**) no STAR control plasmid and (**l**) no sgRNA or no target RNA control plasmid. Constitutive promoters are colored black and inducible promoters are colored purple and labelled accordingly.

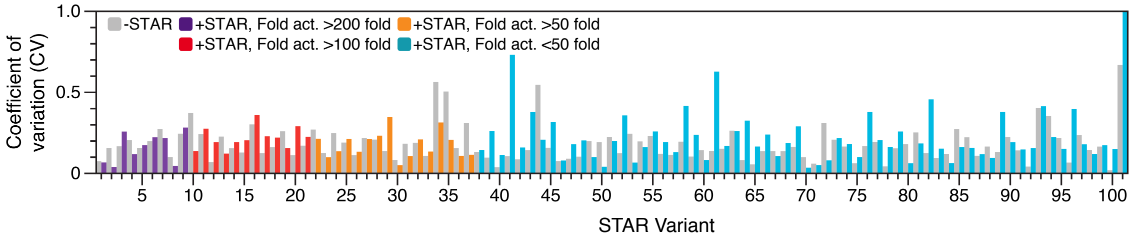

Supplementary Figure 4. Coefficient of variation for fluorescence characterization of STAR variants. Coefficient of variation (CV) was determined by calculating the ratio of the standard deviation and the mean of fluorescence measurements for the STAR:target variants. Data is derived from fluorescence characterization described in **Figure 1b** for both in the absence (–STAR) and the presence (+STAR) of cognate STAR expression plasmids. The y-axis was limited to 1 to aid interpretation. The CV for variant 101 in the +STAR condition was 1.93.

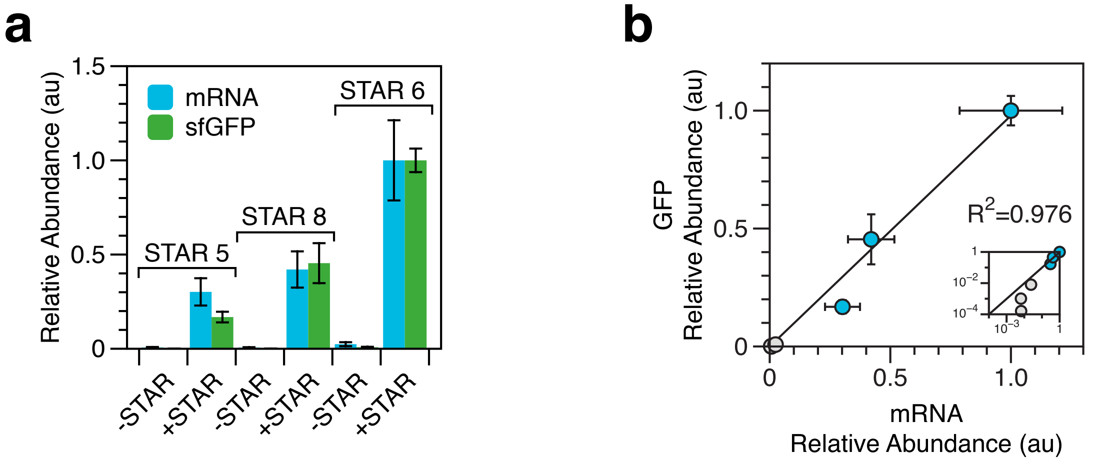

Supplementary Figure 5. Comparison of reverse transcription quantitative PCR (RT-qPCR) and fluorescence characterization of STAR variants. Relative abundance of sfGFP mRNA (mRNA) and protein expression (sfGFP) for three STAR:target variants. (**a**) RT-qPCR (measuring relative abundance of sfGFP mRNA) and fluorescence characterization (measuring relative abundance of sfGFP protein in units of fluorescence/optical density at 600 nm) was performed on *E. coli* cells transformed with different target DNA plasmids in the absence (-STAR) and presence (+STAR) of a DNA plasmid encoding a cognate STAR. (**b**) Correlation of determination (R^2) between RT-qPCR and fluorescence characterization is shown. Data for each type of measurement were normalized to 1 for the +STAR condition of STAR variant 6 and error propagated. Fluorescence data represent mean values and error bars represent s.d. of n = 9 biological replicates. RT-qPCR data represent mean values and error bars represent s.d. of n = 3 biological replicates each quantified with n = 3 technical replicates.

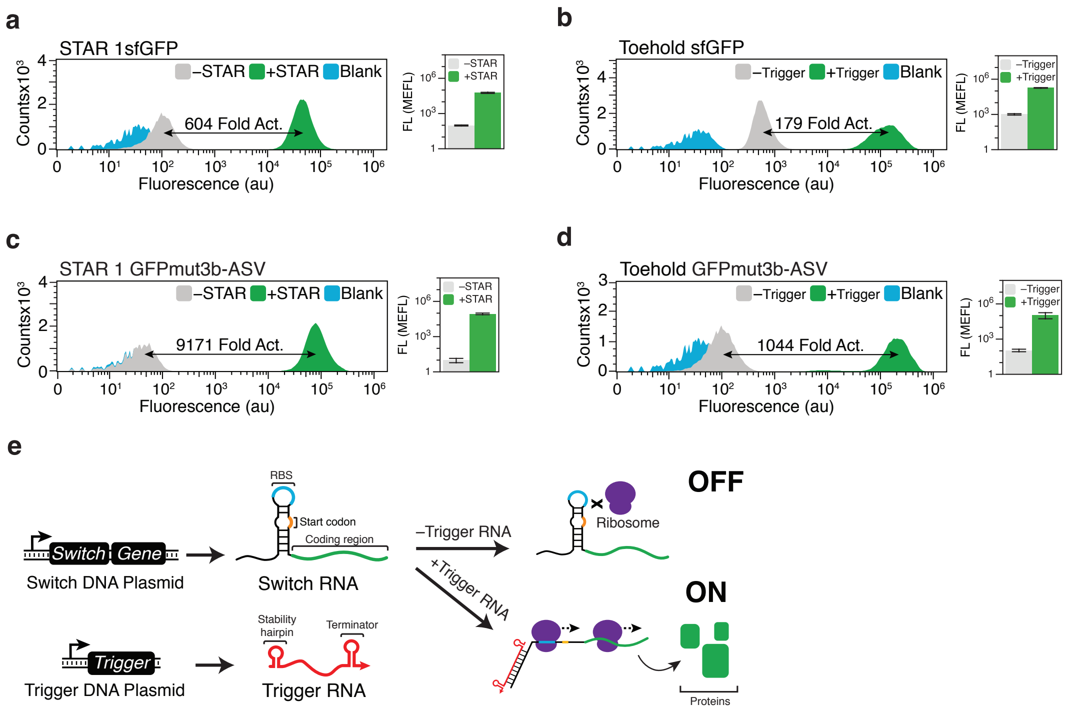

Supplementary Figure 6. Comparison of a STAR and a toehold translational activator. Fluorescence characterization of the best-performing STAR and the best performing toehold translational activator from Green *et al*.[3] Fluorescence characterization performed with both (**a**, **b**) sfGFP and (**c**, **d**) GFPmut3b-ASV. Flow cytometry histograms of a representative biological replicate shown on the left of each panel and graph of mean fluorescence of biological replicates shown on the right of each panel. (**e**) Toehold switches function by designing a switch RNA to form a hairpin structure around the ribosome binding site (RBS) to repress ribosome binding and translation initiation. Activation is achieved by addition of a trigger RNA designed to disrupt hairpin formation and allow translation initiation. The best performing toehold switch (designated forward engineered switch 1 in Green *et al*.)[3] was chosen for characterization within the experimental setup described for STARs. We note several major differences in the characterization experiment performed here and that performed in Green *et al*. including: using *E. coli* RNA polymerase (RNAP) to express trigger and switch RNA (originally T7 polymerase), use of *E. coli* RNAP transcriptional terminators, different DNA plasmid back bones, use of TG1 *E. coli* strain (originally BL21 STAR DE3) and M9 minimal media (originally LB). Data were collected by flow cytometry. Fluorescence characterization (measured in units of arbitrary fluorescence [au] or units of Molecules of Equivalent Fluorescein [MEFL]) was performed on *E. coli* cells transformed with different target DNA plasmids in the absence (–STAR or –Trigger) and presence (+STAR or +Trigger) of a DNA plasmid encoding cognate STAR or Trigger, compared to the autofluorescence of *E. coli* cells transformed with control plasmids (Blank). Representative flow cytometry histograms of n = 1 biological replicates and bar graphs represent mean values and error bars represent s.d. of at least n = 7 biological replicates.

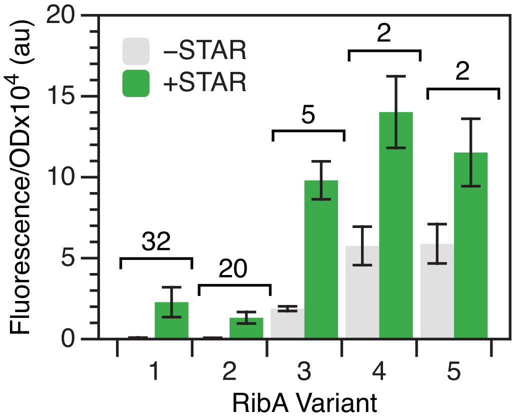

Supplementary Figure 7. Experimental characterization of ribA STARs. Characterization of five STAR:target RNAs utilizing the terminator from the *E. coli ribA* gene. Fluorescence characterization (measured in units of fluorescence/optical density [OD] at 600 nm) was performed with *E. coli* cells transformed with DNA target plasmids in the absence (–STAR) and presence (+STAR) of a DNA plasmid encoding cognate STAR. Data represent mean values and error bars represent s.d. of n = 9 biological replicates.

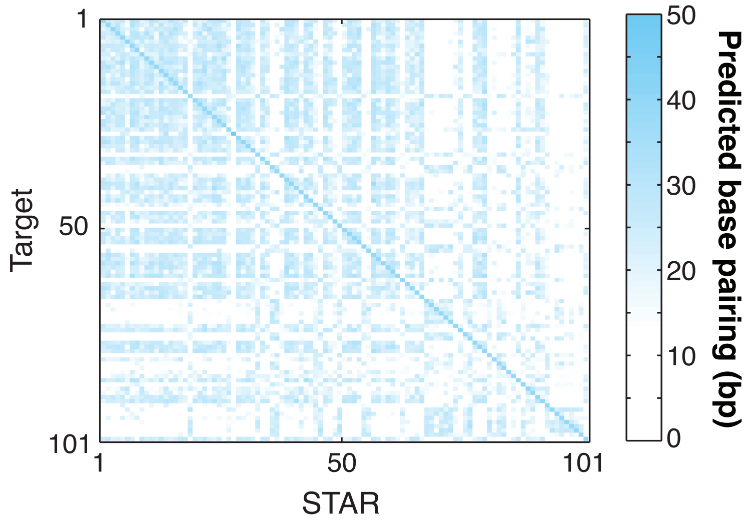

Supplementary Figure 8. Computational prediction of base pairing between STAR and target RNA variants. Computationally predicted base pairing (bp) between the STAR and target RNA variants using NUPACK as described in **Supplementary Note 3**. The matrix shows the predicted pairing for each combination of the 101 STAR:target RNA variants (10,201 combinations).

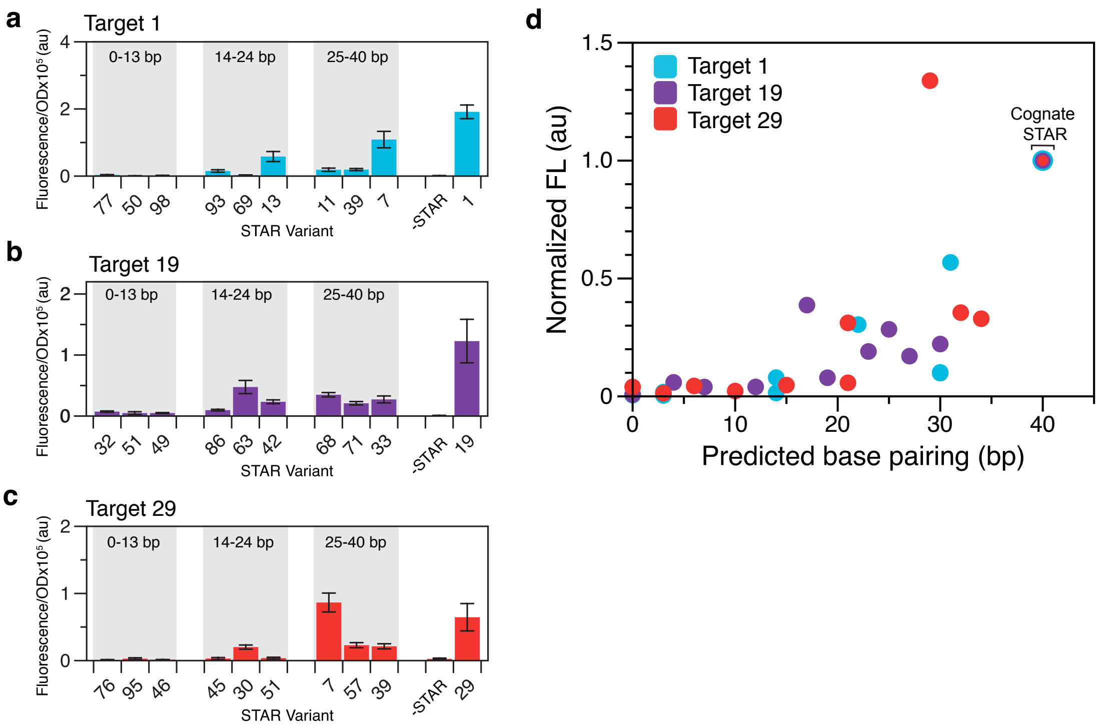

Supplementary Figure 9. Determining the relationship between predicted STAR:target RNA base pairing and orthogonality. (**a-c**) Fluorescence characterization of three target RNAs in combination with 9 non-cognate STARs that were predicted to form between 0-13 base pairs (bp), 14-24 bp or 25-40 bp by the NUPACK analysis algorithm as described in **Supplementary Note 3**. For each target RNA, fluorescence characterization was performed in the absence (-STAR) and presence (+STAR) of a DNA plasmid encoding a STAR variant (indicated below graph) (cognate STAR shown in right most bar in **a-c**). (**d**) Normalized fluorescence of non-cognate and cognate STARs plotted against predicted base pairing. Fluorescence was normalized to the fluorescence value for the cognate STAR. Data represent mean values and error bars represent s.d. of *n* = 9 biological replicates.

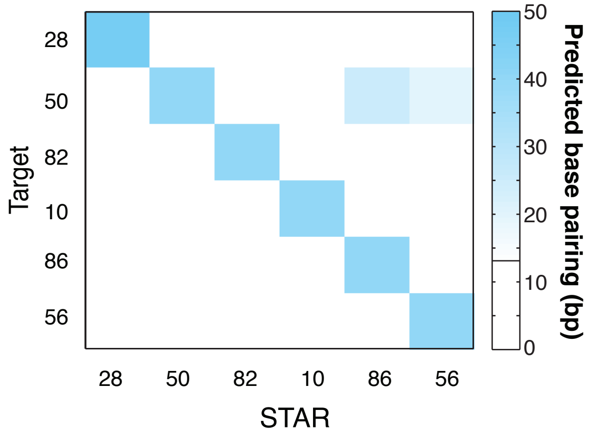

Supplementary Figure 10. Computational prediction of STAR orthogonality. A computationally predicted set of orthogonal STARs was identified using an algorithm described in **Supplementary Note 3**. In this algorithm, STAR:target RNAs were predicted to be orthogonal if base pairing between the linear region and linear binding sequence of a target and STAR was less than 13 bp for non-cognate pairs in one of the two combinations (e.g. either STAR 1:target 2 or STAR 2:target 1). The matrix shows the predicted pairing for each combination of a specific set of STAR:target RNA variants. Less than 13 bp of interaction are colored white and 50 bp of interaction are colored blue. These predictions were validated experimentally through gene expression measurements in *E. coli* cells shown in **Fig. 1d**.

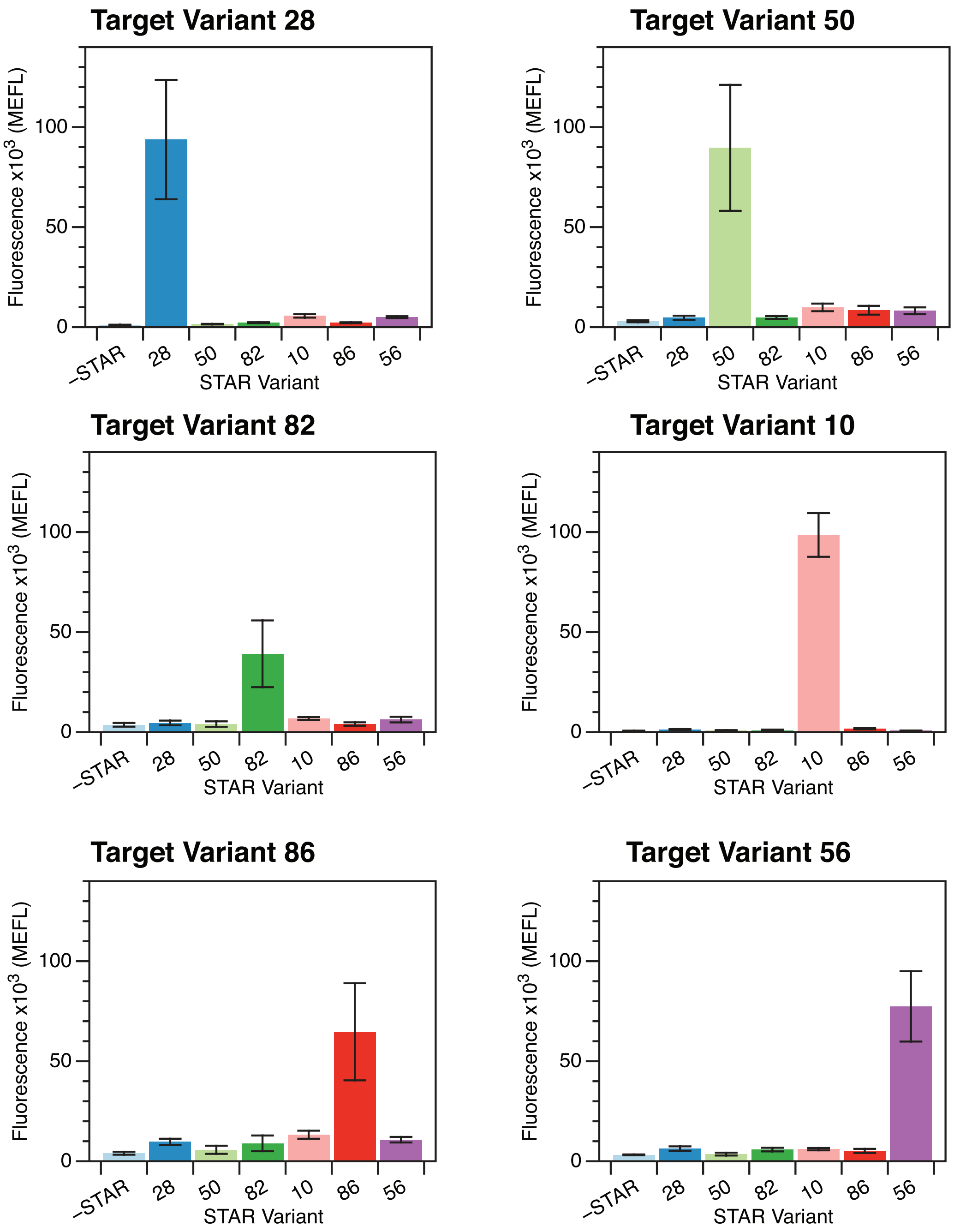

Supplementary Figure 11. Experimental characterization of an orthogonal STAR library. Orthogonality of a 6x6 library of target RNA and STAR variants. Fluorescence characterization (measured in units of Molecules of Equivalent Fluorescein [MEFL]) was performed on *E. coli* cells transformed with different target DNA plasmids (labeled above each panel) in the absence (-STAR) and presence (+STAR) of a DNA plasmid encoding a cognate or non-cognate STAR variant (shown on x-axis of each panel). Data were measured with flow cytometry. Data represent mean values and error bars represent s.d. of at least *n* = 7 biological replicates.

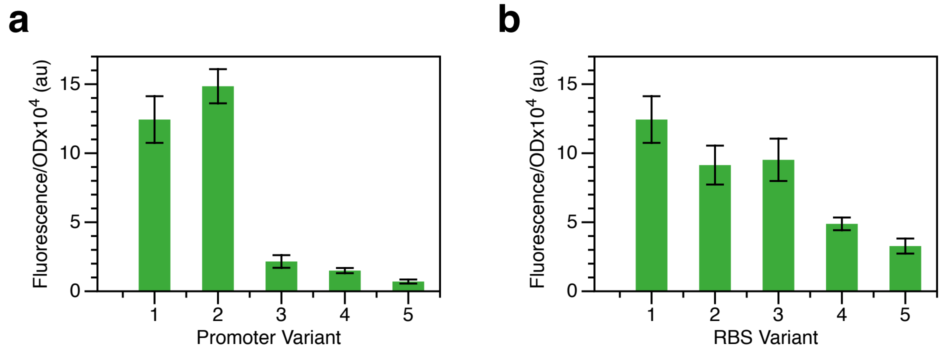

Supplementary Figure 12. Variable strength promoter and RBS library. Characterization of five different strength promoters and ribosome binding sites (RBS) variants (see **Supplementary Table 1** for sequences). Promoter and RBS variants were used to drive expression of a sfGFP containing an RNA stability hairpin derived from a previously published library[2]. (**a**) RBS 1 was used for the promoter library and (**b**) promoter 1 was used in the RBS library. Fluorescence characterization (measured in units of fluorescence/optical density [OD] at 600 nm) was performed on *E. coli* cells transformed with a library of DNA plasmids containing variable strength promoters and RBSs. Data represent mean values and error bars represent s.d. of n = 9 biological replicates.

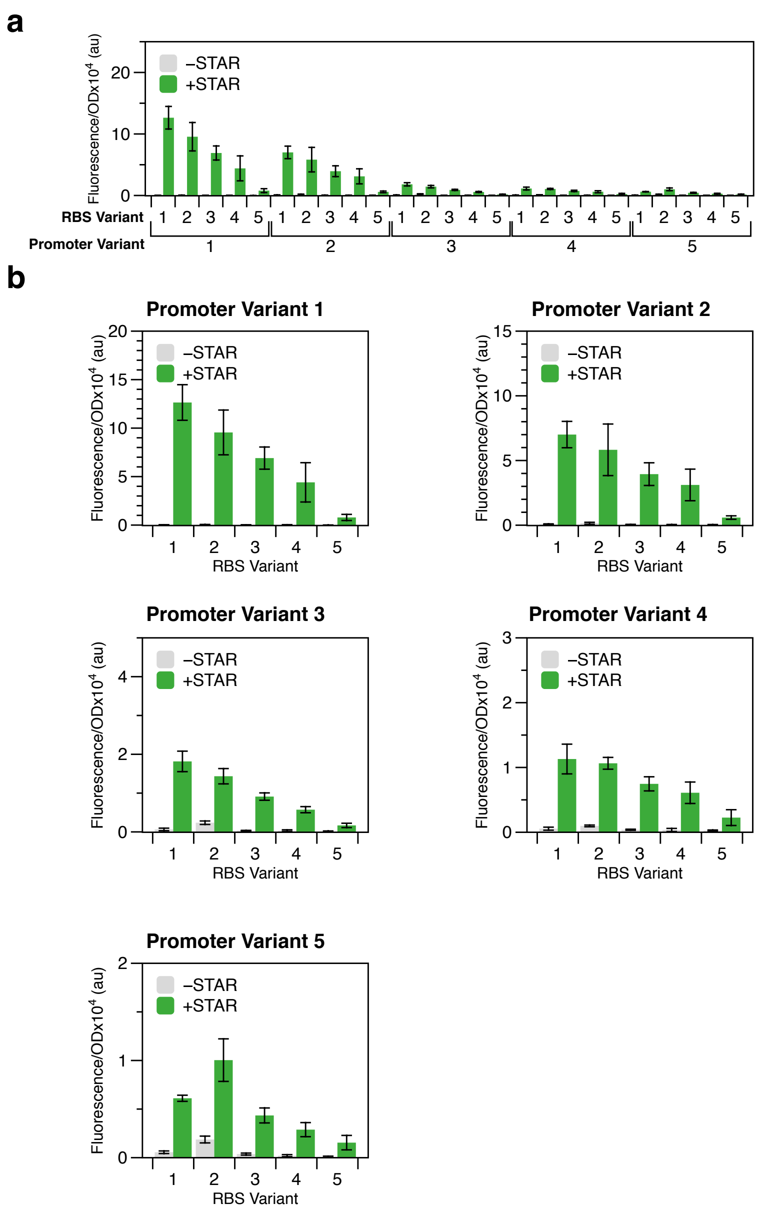

Supplementary Figure 13. Creating switchable promoter and ribosome binding site strength libraries with STARs. Target variant 5 was combined with 5 different strength promoters and ribosome binding sites (RBS) variants (see **Supplementary Table 1** for sequences). Promoters and RBSs variants are numbered 1-5 from strongest to weakest. Fluorescence characterization (measured in units of fluorescence/optical density [OD] at 600 nm) was performed on *E. coli* cells transformed with a library of target DNA plasmids containing variable strength promoters and RBSs in the absence (-STAR) and presence (+STAR) of a DNA plasmid encoding STAR variant 5. (**a**) Full library and (**b**) each promoter variant are shown in panels with RBS variants shown on the x-axis. Data represent mean values and error bars represent s.d. of n = 9 biological replicates.

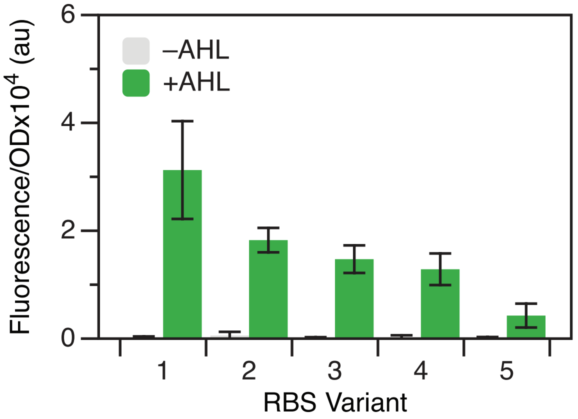

Supplementary Figure 14. Combining the inducible promoter-STAR system with the target RNA RBS strength library. The inducible promoter-STAR system was combined with the target RNA-RBS strength library and assayed for function. Fluorescence characterization (measured in units of fluorescence/optical density [OD] at 600 nm) was performed on *E. coli* cells transformed with DNA target-RBS library plasmids in the absence (–AHL) and presence (+AHL) of 100 nM AHL. Data represent mean values and error bars represent s.d. of *n* = 9 biological replicates.

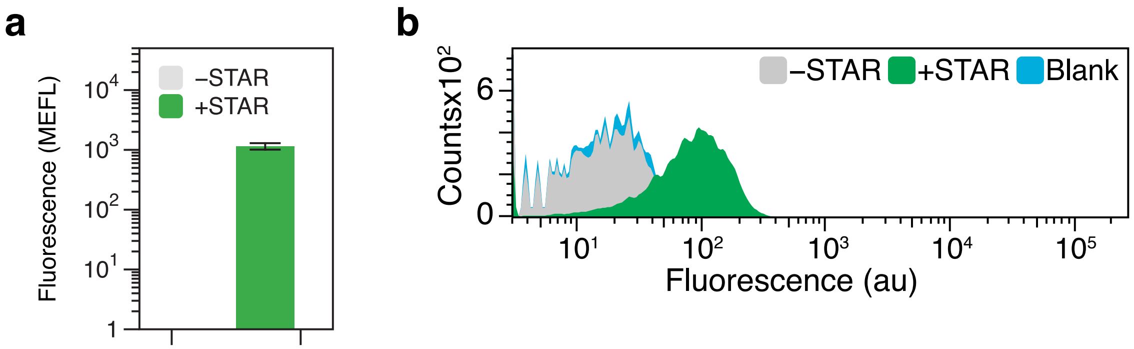

Supplementary Figure 15. STARs regulate sfGFP expression from target expression cassettes integrated into the *E. coli* genome. A STAR variant 5 regulated *sfgfp* gene was integrated into the genome of *E. coli* TG1 cells to create *E. coli* TG1 target variant 5 *sfgfp* (**Supplementary Table 3**)**.** Fluorescence characterization was performed on *E. coli* TG1 target variant 5 *sfgfp* cells in the absence (-STAR) and presence (+STAR) of a DNA plasmid encoding STAR variant 5, and compared to the autofluorescence of *E. coli* cells transformed with control plasmids (Blank). Data were collected by flow cytometry. Data in (**a**) represent mean fluorescence (measured in units of Molecules of Equivalent Fluorescein [MEFL]) values and error bars represent s.d. of at least n = 7 biological replicates. (**b**) A representative flow cytometry histogram of n = 1 biological replicates (measured in units of arbitrary fluorescence [au]).

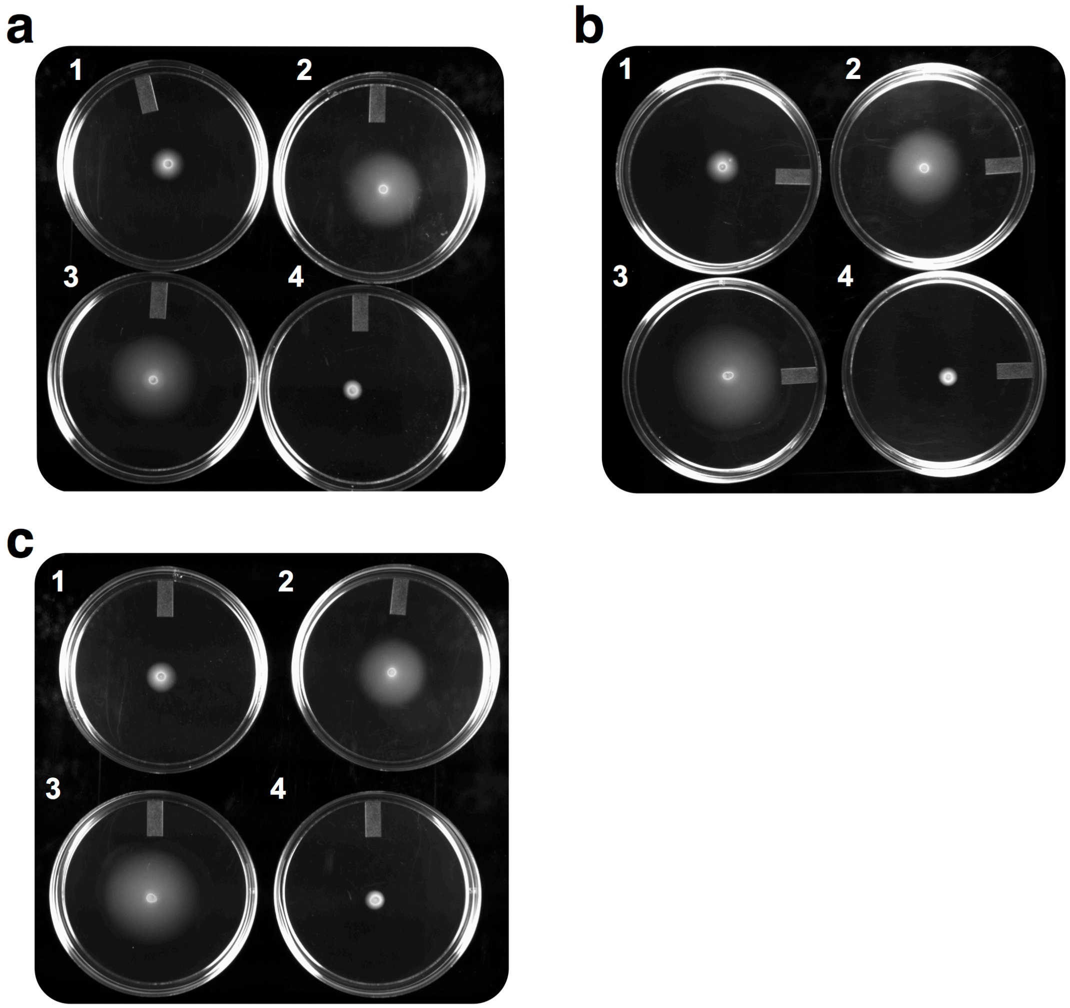

Supplementary Figure 16. Controlling cell motility with STARs. A STAR variant 5 regulated *cheZ* gene was integrated into the genome of *E. coli* BW25113Δ*cheZ* cells to create *E. coli* BW25113Δ*cheZ* target variant 5 *cheZ* (**Supplementary Table 3**). Photographs of semi-solid motility assays of *E. coli* BW25113Δ*cheZ* target variant 5 *cheZ* cells in the absence (1) and presence (2) of a DNA plasmid encoding STAR variant 5. The parent strain *E. coli* BW25113 (3) and *E. coli* BW25113Δ*cheZ* (4) cells transformed with control plasmids were used as motile and non-motile controls. Each photograph show results from *n* = 1 biological replicate performed independently (**a**-**c**).

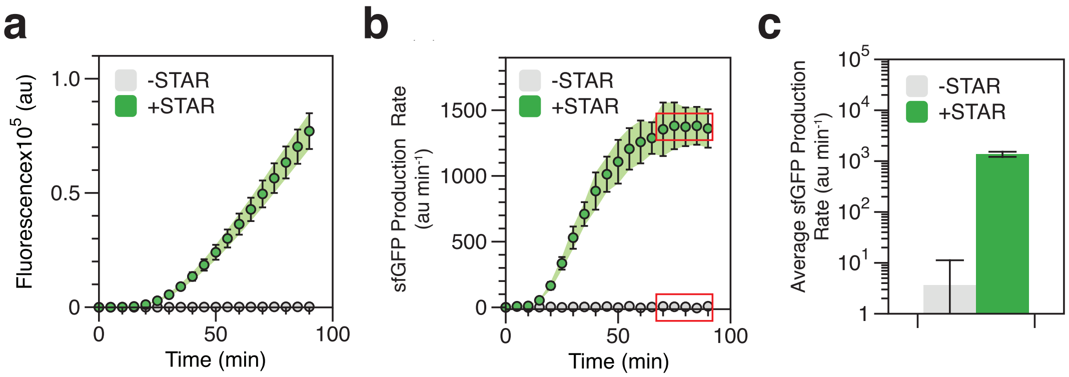

Supplementary Figure 17. Characterization of STARs in cell-free transcription and translation (TX-TL) reactions. Characterization of STAR variant 5 expressing sfGFP in TX-TL reactions. (**a**) Fluorescence characterization (measured in units of arbitrary fluorescence [au]) performed in TX-TL reactions containing 8 nM of target DNA plasmid variant 5 and either 15 nM of a no-STAR control plasmid (-STAR) or 15 nM of a DNA plasmid encoding STAR variant 5 (+STAR). (**b**) The production rate of sfGFP (measured in units of au minute^{-1}) was determined by taking the time derivative of (**a**). (**c**) The average sfGFP production rate was determined within the linear phase of sfGFP production rate highlighted in the red box in (**b**). Data represent mean values and error bars represent s.d. of n = 9 biological replicates.

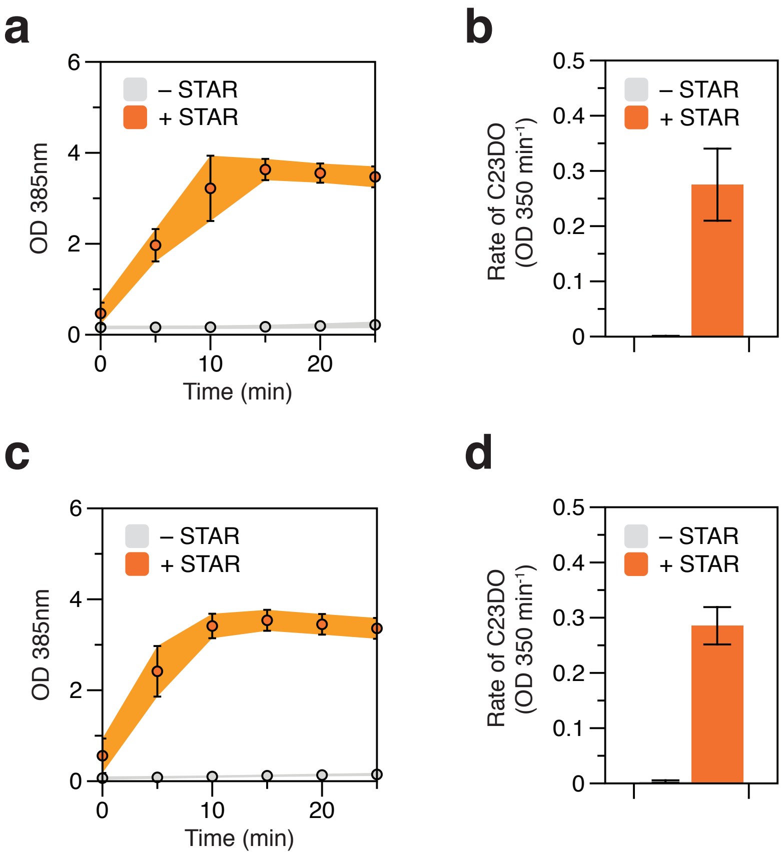

Supplementary Figure 18. Characterization of catechol 2,3-dioxygenase (C23DO) enzyme expression for one-to-one and one-to-many regulation. STAR controlled expression of C23DO in *E. coli* cells for (**a**, **b**) one-to-one and (**c**, **d**) one-to-many regulation. (**a**, **c**) Kinetic spectral characterization (measured in units of OD at 385 nm) was used to determine the (**b**, **d**) rate of C23DO conversion of catechol to 2-hydroxymuconate semialdehyde between 0 and 10 minutes. C23DO expression characterization was performed on *E. coli* cells transformed with either (**a**, **b**) a DNA plasmid encoding target RNA regulated C23DO alone or (**c**, **d**) DNA plasmids encoding target RNA regulated C23DO, mRFP and sfGFP simultaneously. Data represent mean values and error bars represent s.d. of n = 9 biological replicates.

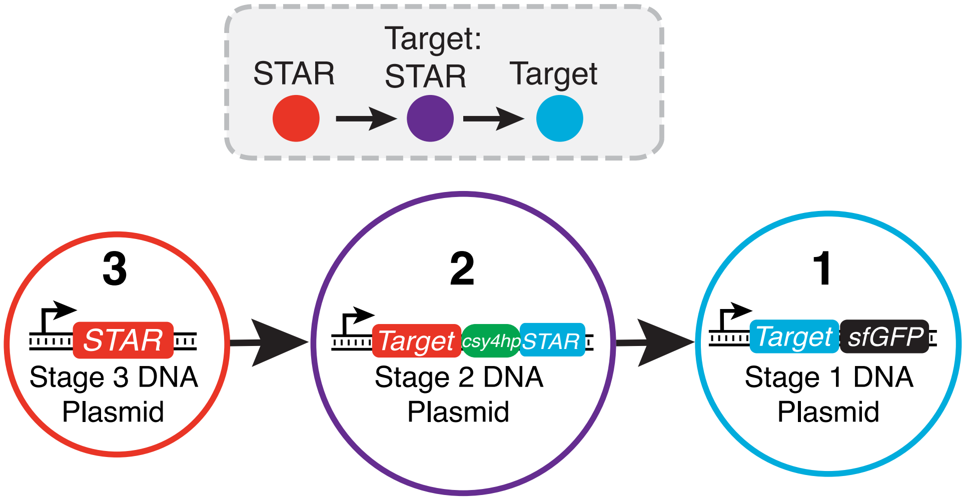

Supplementary Figure 19. Schematic of the STAR activation-activation cascade. The activation-activation cascade is composed of three stages. Stage 3 is a STAR that activates its cognate target RNA on stage 2. This target RNA is transcriptionally fused to a Csy4 hairpin (Csy4 hp)[8] and an orthogonal STAR. A heterologously expressed Csy4 gene cleaves[8] the Csy4 hp, releasing the orthogonal STAR from the target RNA, allowing it to activate its cognate target RNA on stage 1 that is transcriptionally fused to an sfGFP gene. sfGFP should only be expressed in the presence of the full cascade.

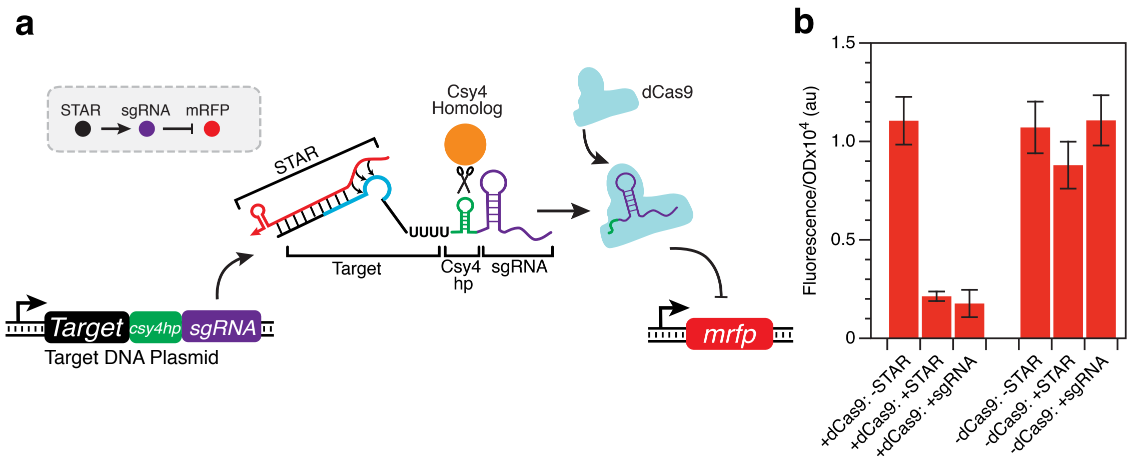

Supplementary Figure 20. Characterization of STAR regulated CRISPR interference (CRISPRi). (**a**) Schematic of STAR regulated CRISPRi. The STAR regulates expression of a target RNA-Csy4 hairpin-single guide RNA (sgRNA) transcriptional fusion. A Csy4 homolog endogenous to *E. coli* can cleave the Csy4 hairpin (Csy4 hp)[8], releasing the sgRNA to form a complex with a catalytically dead mutant of the Cas9 protein (dCas9) that binds to the *mrfp* DNA sequence to repress transcription. The mRFP expression cassette is located on the STAR expressing plasmid or no-STAR control plasmid. (**b**) Fluorescence characterization (measured in units of fluorescence/optical density [OD] at 600 nm) was performed on *E. coli* cells transformed with a STAR variant 5 regulated sgRNA DNA plasmid in the absence (-STAR) or presence (+STAR) of a DNA plasmid encoding STAR variant 5. A DNA plasmid encoding a constitutively expressed sgRNA (+sgRNA) with no Csy4 hairpin was used as a positive control of repression. These conditions were performed in the absence (-dCas9) and the presence (+dCas9) of a DNA plasmid encoding the dCas9 protein. Data represent mean values and error bars represent s.d. of *n* = 9 biological replicates.

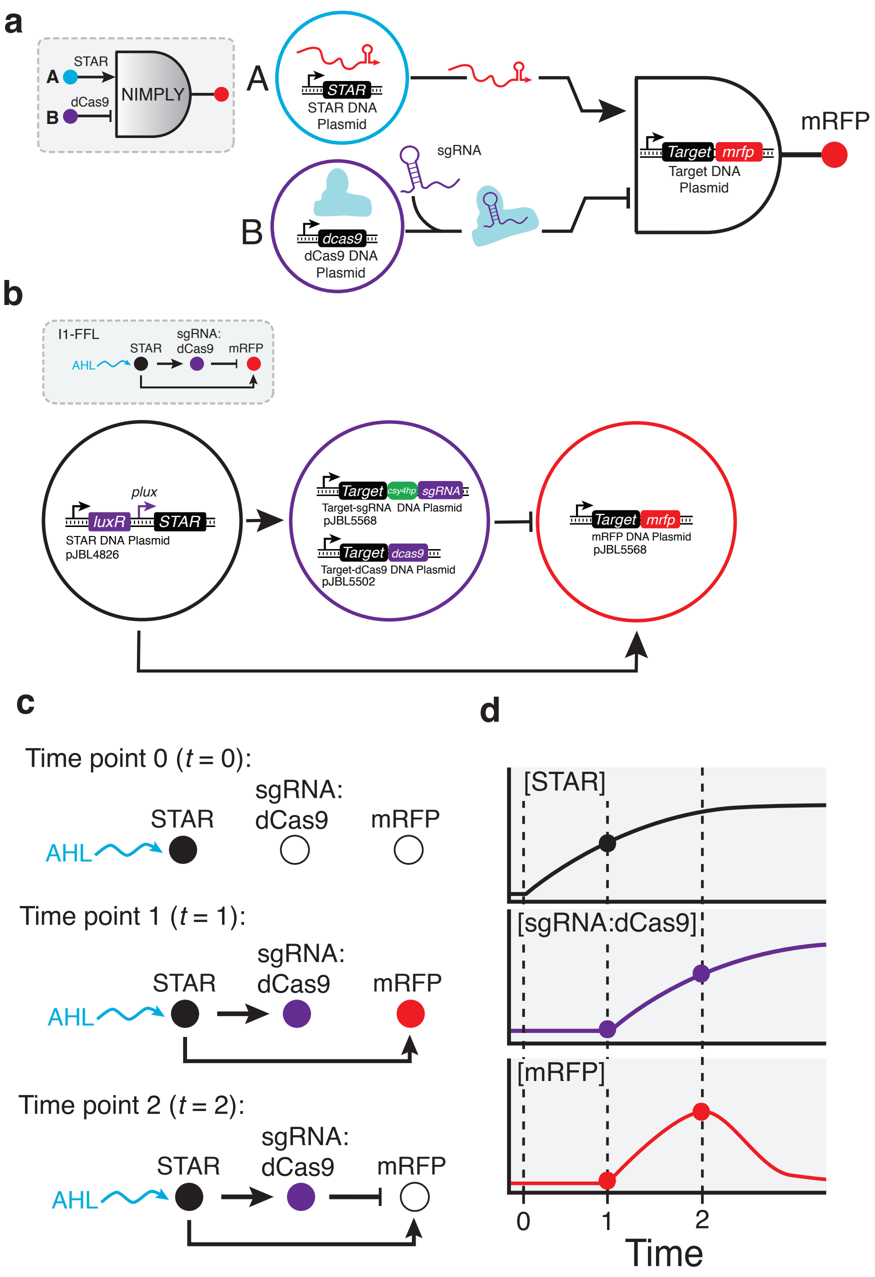

Supplementary Figure 21. Schematic of the STAR and CRISPRi genetic circuitry. (**a**) Schematic of STAR and CRISPRi NIMPLY (A AND NOT B) logic gate. Input A is a STAR that activates transcription of a target RNA regulated *mrfp* gene. Input B is a catalytically dead mutant of the Cas9 protein (dCas9) that in combination with a constitutively expressed single guide RNA (sgRNA) represses transcription of the *mrfp* gene. Only in the presence of input A and in the absence of input B will mRFP be expressed. (**b**) Schematic of DNA plasmids for the Incoherent Type 1 Feed Forward Loop (I1-FFL). (**c**) Schematic of temporal signal propagation through a STAR and CRISPRi I1-FFL[9] and (**d**) cartoon graphs of the resulting levels of I1-FFL species over time. AHL is added

at $t = 0$ to induce STAR expression. STAR accumulates and at $t = 1$ an activation threshold is reached to activate mRFP transcription, resulting in an increase in mRFP levels. Simultaneously the sgRNA and dCas9 expression is activated and at $t = 2$ reaches a repression threshold that represses transcription of mRFP, resulting in decrease in mRFP levels. As a result the I1-FFL creates a pulse of mRFP expression[9]. In addition if mRFP is not completely repressed by CRISPRi, the I1-FFL should accelerate the response time towards steady-state compared to direct activation[9].

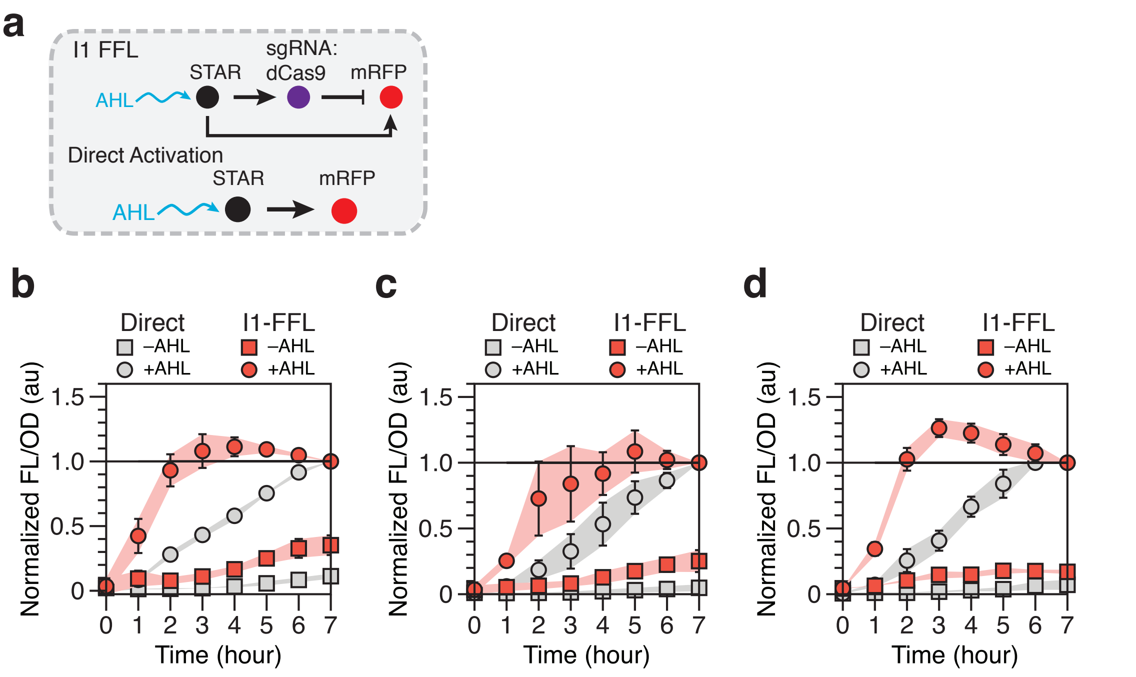

Supplementary Figure 22. Characterization of a STAR and CRISPRi incoherent type 1 feed-forward loop (I1-FFL). (**a**) Schematic of STARs and CRISPRi I1-FFL and direct activation circuits. (**b-d**) Fluorescence characterization (measured in units of fluorescence/optical density [OD] at 600 nm) was performed on *E. coli* cells transformed with plasmids encoding the I1-FFL or the direct activation (direct) cascade. At time 0 hour either acyl-homoserine lactone (+AHL) or water (-AHL) were added and fluorescence measured every 1 hour for 7 hours. Fluorescence data for each circuit were individually normalized by dividing by the final fluorescence values at 7 hours in the +AHL condition for each colony before calculating the mean and standard deviation at each time point. (**b-d**) Each panel represent mean values and error bars represent s.d. of *n* = 3 biological replicates collected for independent repeats for comparison.

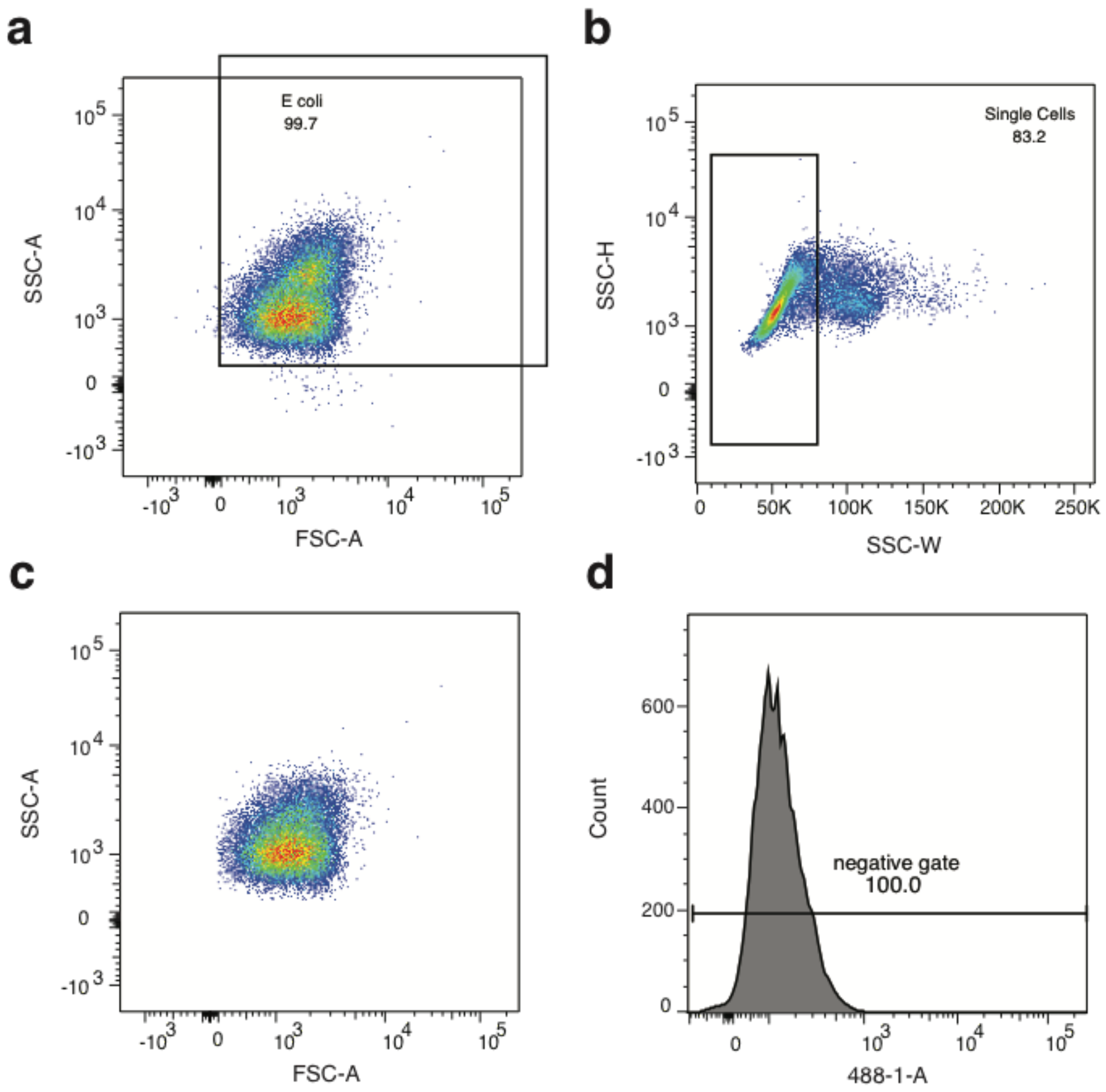

Supplementary Figure 23. Flow cytometry gating. (**a**) *E. coli* gate based upon side scatter (SSC-A) and forward scatter (FSC-A) for an *E. coli* culture. (**b**) Single cell gate based upon side scatter pulse height (SSC-H) and side scatter pulse width (SSC-W). (**c**) Resulting population from *E. coli* gate and single cell gates combined. (**d**) Negative gate based upon fluorescence. See **Supplementary Note 4** for detailed description of gating used.

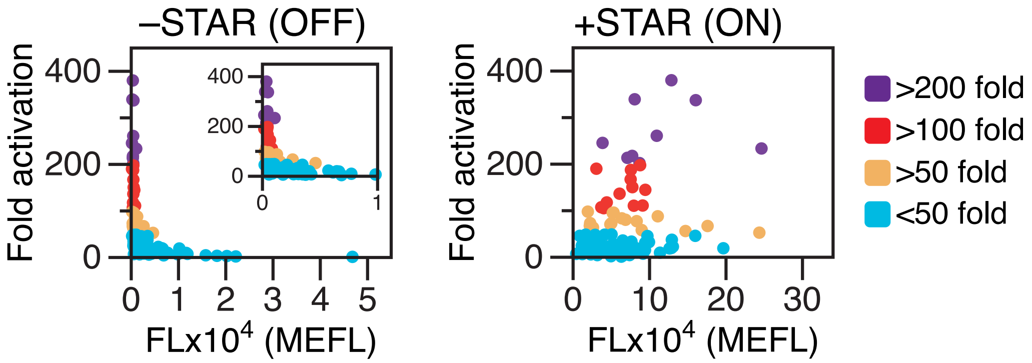

Supplementary Figure 24. STAR:target RNA characteristics governing dynamic range. Fluorescence characterization of each target RNA in the absence (–STAR) (left panel) and presence (+STAR) of cognate STAR (right panel) relative to fold of activation. Each target RNA variant is colored accordingly to the fold of activation. Fluorescence characterization data from **Figure 1b**. We note that STAR:target variant 101 was not considered because no activation was observed.

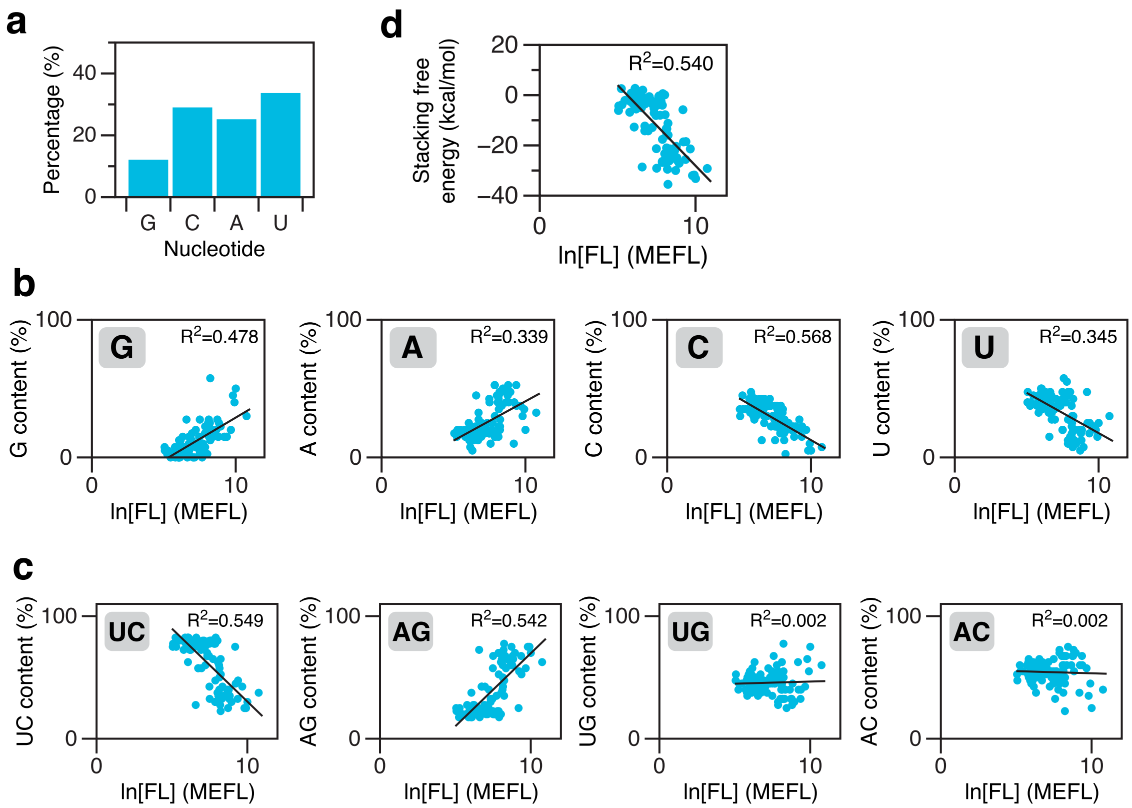

Supplementary Figure 25. Relationship between target RNA sequence composition, base stacking free energy and transcriptional termination efficiency. (**a**) Percentage nucleotide composition for the 100 computationally designed target RNA linear regions. (**b**) Relationship between the percentage content of single nucleotide (nucleotide identity shown in upper left of graph) or (**c**) double nucleotides (identity shown in upper left of graph) of the different target RNA linear regions and the natural log of fluorescence in the absence of STAR (OFF state). (**d**) Base stacking free energy of the different target RNA linear regions and the natural log of fluorescence in the absence of STAR (OFF state). Base stacking free energies were calculated using dinucleotide base stacking free energies previously determined[10]. Line of best fit is plotted and the correlation of determination (R^2) is displayed in upper right of graph. Natural log of fluorescence characterization data from **Fig. 1b**. We note that STAR:target variant 101 was not considered because no activation was observed.

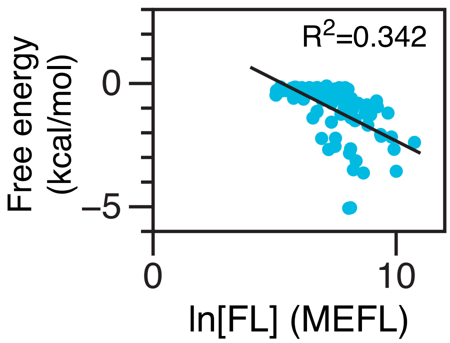

Supplementary Figure 26. Relationship between predicted target RNA secondary structure and transcriptional termination efficiency. Secondary structure ensemble free energy of the different target RNA linear regions was predicted using NUPACK[11] and plotted against the natural log of fluorescence in the absence of STAR (OFF state). Line of best fit is plotted and the correlation of determination (R^2) is displayed in upper right of graph. Natural log of fluorescence characterization data from **Fig. 1b**. We note that STAR:target RNA variant 101 was not considered because no activation was observed.

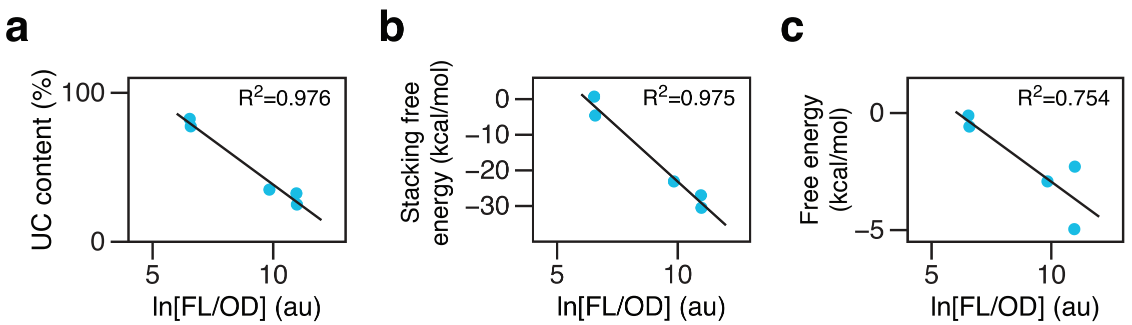

Supplementary Figure 27. Relationship between transcriptional termination efficiency and sequence composition, base stacking free energy and predicted secondary structure of the ribA target RNAs. (**a**) Percentage content of uracil (U) and cytosine (C) nucleotides in the different target RNA linear regions and the natural log of fluorescence in the absence of STAR (OFF state). (**b**) Base stacking free energy of the different target RNA linear regions and the natural log of fluorescence in the absence of STAR (OFF state). Base stacking free energies were calculated using dinucleotide base stacking free energies previously determined[10]. (**c**) Ensemble free energy of the different target RNA linear regions was predicted using NUPACK[11] and plotted against the natural log of fluorescence in the absence of STAR (OFF state). Natural log of fluorescence characterization data from **Supplementary Fig. 7**.

Supplementary Note 1. Computational design of STARs using the NUPACK design tool

To computationally design STAR variants, we harnessed the NUPACK RNA design algorithm[7]. To do this we used the NUPACK design web-application[6], running the NUPACK design script below. In brief, the script designs RNA sequences for the linear binding sequence and linear region of the STAR and target RNA, respectively, according to the design schematic in **Supplementary Fig. 2**. In this case, NUPACK designs STAR and target RNAs that are unstructured on their own but form a strong heteroduplex when present together. Certain sequences are prevented in designs and defects within the structure are allowed.

Designing

```
# STAR computational design script for NUPACK web-application

material = rna
temperature = 37.0
trials = 3
sodium[M] = 1.0
dangles = some
allowmismatch = true

structure STAR = .............................................................

structure Target =
......................................................................................

structure ON =
(((((((((((((((((((((((((((((((((((((((((((((((((((((((((((((+))))))))))))))))))))))))))))
)))))))))))))))))))))))))))))))))).........................


domain a = N40

domain b = GCGGGGAATGTATACAGTTCATGTATATATTCCCCGCTTTTTTTTT

domain c = TGAACTGTATACATTCCCCGC


STAR.seq = c a*

Target.seq = a b

ON.seq = c a* a b

prevent = AAAA, CCCC, GGGG, UUUU, KKKKKK, MMMMMM, RRRRRR, SSSSSS, WWWWWW, YYYYYY

STAR.stop = 30.0
```

Supplementary Note 2. Determining STAR design principles

Our goal was to determine whether we could uncover design principles from the computationally designed STAR library. We began by understanding the relationship between target RNA regulatory characteristics and dynamic range. We first compared the fluorescence characterization of our target RNA library in both the absence (-STAR [OFF state]) and presence (+STAR [ON state]) of STAR, to the fold activation of each STAR:target variant (**Supplementary Fig. 24**). We note that variant 101 was excluded because this design gave rise to no activation (**Figure 1b**). While we saw a greater variation in the ON state fluorescence for designs with high-dynamic range, we observed that designs with high-dynamic ranges consistently had a low OFF level of fluorescence. In other words, target RNA transcriptional termination efficiency in absence of STAR appeared to be a key determinant of dynamic-range.

Given this, we next aimed to determine the relationship between target RNA sequence and structure, and the OFF state fluorescence, which we used as a proxy for transcriptional termination efficiency. We began by studying the effect of sequence composition of the computationally designed linear region of the target RNA (**Supplementary Fig. 25**). Overall we observed a relatively even distribution of the four nucleotides across the computationally designed target RNAs, with a slight decreased preference for guanosine (**Supplementary Fig. 25a**). We next compared how percentage content of each nucleotide affected OFF state fluorescence. We note that as percentage nucleotide content is often used as a proxy for free energy, we compared this to the natural log of fluorescence according to a model whereby the observed fluorescence is proportional to the equilibrium constant between the folded and un-folded states of the linear region. This model effectively assumes equilibration of the linear region before termination, which could be valid given the fast timescales of RNA folding and the timescale of pausing of polymerase on a polyU tract. Interestingly we observed a relatively strong negative correlation (R^2 of ~0.5) between both uracil (U) and cytosine (C) percentage content and the OFF state fluorescence (**Supplementary Fig. 25b**). Moreover, we observed a similar correlation when both U and C content were combined to give an R^2 of ~0.5 (**Supplementary Fig. 25c**). Taken together, this suggests that high UC content improves transcription termination efficiency of the target RNA. We hypothesized that this bias towards UC pyrimidine nucleotides for high termination efficiency may be attributed to base stacking interactions. Stacking interactions between the aromatic nucleotide bases are a major contributor to RNA structures. For example, single-stranded RNAs base stacking has been shown to influence structural properties such as rigidity and formation of partial helical conformations[12]. Moreover, it is well-established that sequence is a major determinant of base stacking interactions, with decreasing stacking free energies in the order purine-purine, purine-pyrimidine, pyrimidine-purine and pyrimidine-pyrimidine[10]. Indeed, when we calculate the stacking free energy of different target RNA's linear regions

according to previously determined stacking free energy values[10], we observed a negative correlation to OFF state fluorescence (**Supplementary Fig. 25d**).

We next turned to the relationship between secondary structure and termination efficiency. Using NUPACK to predict secondary structure within the target RNA's linear region we compared the predicted ensemble free energy of each target RNA to the OFF state fluorescence (**Supplementary Fig. 26**). We observed a negative correlation with an R^2 of 0.342, suggesting that secondary structure within the linear region of the target RNA negatively impacts the transcription termination efficiency.

Taken together this suggested that both the presence of base stacking and secondary structure within the target RNA's linear region decreased transcription termination efficiency. We next sought to determine whether these observations represented a general STAR design principle or were specific for the AD1 terminator hairpin that was used as a terminator scaffold for the STAR library. To test this, we used NUPACK to computationally design a small library of STARs using the terminator from the *E. coli ribA* gene as a terminator scaffold[5]. This library was constructed and functionally characterized (**Supplementary Fig. 7**). We again observed a strong negative correlation between the OFF state fluorescence and UC content (R^2 0.976), stacking free energy (R^2 0.975) and ensemble free energy (R^2 0.754) of the target RNA's linear region (**Supplementary Fig. 27**). As such, this suggested that the negative impact of base stacking and secondary structures on transcription termination efficiency appeared to be a generalizable principle for the STAR regulatory system.

Supplementary Note 3. Predicting orthogonal STAR libraries

To predict orthogonal STAR:target RNA pairs, we developed an in house algorithm that uses NUPACK[13] to model STAR:target interactions and select pairs with minimal interactions between non-cognate pairs. This algorithm first creates two NUPACK input files for each of the 101 target RNAs: `<prefix>.in` and `<prefix>.list` whereby <prefix> is the target variant identifier. The `<prefix>.in` file specifies the number of strands (101 STARs and 1 target RNA), the 101 STAR variant sequences and the specific target variant sequence, as shown below:

```
<prefix>.in file structure:
102 # number of strands
NNNNNNN # STAR variant 1 sequence
NNNNNNN # STAR variant 2 sequence
…
NNNNNNN # Target variant 1 sequence
1 # option not used
```

It should be noted that only the linear binding sequences and linear region of the STAR:target were used.

The `<prefix>.list` file specifies the strand composition of the complexes to be analyzed – in this case all the STAR variants against a single target RNA.

```
<prefix>.list file structure:
1 102 # Complex of sequence 1 and sequence 102 in <prefix>.in
(STAR variant 1 and target variant 1)
2 102 # Complex of sequence 2 and sequence 102 in <prefix>.in
(STAR variant 2 and target variant 1)
….
101 102 # Complex of sequence 101 and sequence 102 in <prefix>.in
(STAR variant 101 and target variant 1)
```

The partition function, equilibrium base-pairing probabilities and minimum free energy (MFE) structures of the STAR-target complexes are then calculated by running NUPACK locally using the test tube analysis complex function[7, 11] with the following options:

```
complexes -T 37 -material rna -pairs -mfe —degenerate <prefix>
```

This results in an output file called `<prefix.ocx-mfe> which` contained the predicted minimum free energy folds and a dot-bracket structure for each STAR-target complex folded. The algorithm then compiles the dot-bracket structures of each STAR-target complex and counts the nucleotides that are unpaired or involved in intramolecular structures within the STAR strand. This results in a count of unpaired nucleotides for all possible 10,201 complexes, from which the number of linear region predicted base pairing interactions between STAR and target are determined (**Supplementary Fig. 8**). Based upon experimental

characterization (**Supplementary Fig. 9**) we predicted that sets of STAR:target RNAs that showed less than 13 base pairs of interaction in the target RNA for one of the combinations (i.e. either STAR 1:target 2 or STAR 2:target 1) would be orthogonal. To identify predicted orthogonal sets, we first sorted the list of pair interactions to identify pairs that were predicted to have less than 13 bases of interaction. Additional STAR:target combinations were added to each pair to identify combinations of three STAR:targets predicted to be orthogonal. This was repeated to identify sets of four, five, etc. pairs of orthogonal STAR:targets. An example is shown in **Supplementary Fig. 10** that shows a set of 6 STARs that were identified using this approach, and then experimentally validated (**Figure 1d**).

Supplementary Note 4. Flow cytometry gating

Flow cytometry data collection was performed as explained in the methods. FlowJo (v10.2) software was used for analysis of flow cytometry data. The three gates shown in **Supplementary Fig. 23** were used for all samples. The first gate (*E. coli* gate) is based upon side scatter (SSC-A) and forward scatter (FSC-A) to gate for *E. coli* cells and to remove readings from small debris (**Supplementary Fig. 23a**). The second gate (single cells gate) was used to gate away multicellular aggregations so that only single cells remain (**Supplementary Fig. 23b**). This gate was based upon side scatter pulse height (SSC-H) and side scatter pulse width (SSC-W) and the resulting population shown in **Supplementary Fig. 23c**. The final gate (negative gate) based upon fluorescence was within the second gate and was used to remove any high negative values that can arise from fluorescence baseline subtraction error during data acquisition (**Supplementary Fig. 23d**). We note that the following variants 41, 42, 81, 90, 92 showed some level of bimodality in either the absence or presence of STAR. All other variants were unimodal.

Supplementary References

1. Mutalik VK*, et al.* Precise and reliable gene expression via standard transcription and translation initiation elements. *Nature methods* **10**, 354-360 (2013).
2. Carrier TA, Keasling JD. Library of synthetic 5' secondary structures to manipulate mRNA stability in Escherichia coli. *Biotechnol Prog* **15**, 58-64 (1999).
3. Green AA, Silver PA, Collins JJ, Yin P. Toehold switches: de-novo-designed regulators of gene expression. *Cell* **159**, 925-939 (2014).
4. St-Pierre F, Cui L, Priest DG, Endy D, Dodd IB, Shearwin KE. One-step cloning and chromosomal integration of DNA. *Acs Synth Biol* **2**, 537-541 (2013).
5. Chappell J, Takahashi MK, Lucks JB. Creating small transcription activating RNAs. *Nature chemical biology* **11**, 214-220 (2015).
6. Zadeh JN*, et al.* NUPACK: Analysis and design of nucleic acid systems. *Journal of computational chemistry* **32**, 170-173 (2011).
7. Wolfe BR, Porubsky NJ, Zadeh JN, Dirks RM, Pierce NA. Constrained Multistate Sequence Design for Nucleic Acid Reaction Pathway Engineering. *J Am Chem Soc* **139**, 3134-3144 (2017).
8. Qi L, Haurwitz RE, Shao W, Doudna JA, Arkin AP. RNA processing enables predictable programming of gene expression. *Nat Biotechnol* **30**, 1002-1006 (2012).
9. Alon U. Network motifs: theory and experimental approaches. *Nat Rev Genet* **8**, 450-461 (2007).
10. Jafilan S, Klein L, Hyun C, Florian J. Intramolecular base stacking of dinucleoside monophosphate anions in aqueous solution. *J Phys Chem B* **116**, 3613-3618 (2012).
11. Dirks RM, Bois JS, Schaeffer JM, Winfree E, Pierce NA. Thermodynamic analysis of interacting nucleic acid strands. *Siam Rev* **49**, 65-88 (2007).
12. Seol Y, Skinner GM, Visscher K, Buhot A, Halperin A. Stretching of homopolymeric RNA reveals single-stranded helices and base-stacking. *Phys Rev Lett* **98**, 158103 (2007).
13. Zadeh JN, Wolfe BR, Pierce NA. Nucleic acid sequence design via efficient ensemble defect optimization. *Journal of computational chemistry* **32**, 439-452 (2011).
