# Supplementary material for: Computational design of small transcription activating RNAs for versatile and dynamic gene regulation
Source: Nat Commun. 2017 Oct 19;8:1051. doi: 10.1038/s41467-017-01082-6 (PMC5648800; doi:10.1038/s41467-017-01082-6)
Supplement: Supplementary file 3 — Description of Additional Supplementary Files [file 41467_2017_1082_MOESM3_ESM.pdf]

Description of Additional Supplementary Files

File Name: Supplementary Data 1
Description: Plasmids used in this study.

File Name: Supplementary Data 2
Description: Examples of DNA plasmid sequences

File Name: Supplementary Data 3
Description: Sequences of targets RNAs used in this study

File Name: Supplementary Data 4
Description: Sequences of STARs used in this study
