# Supplementary material for: Computational design of small transcription activating RNAs for versatile and dynamic gene regulation
Source: Nat Commun. 2017 Oct 19;8:1051. doi: 10.1038/s41467-017-01082-6 (PMC5648800; doi:10.1038/s41467-017-01082-6)
Supplement: Supplementary file 2 — Peer Review File [file 41467_2017_1082_MOESM2_ESM.pdf]

Editorial Note: This manuscript has been previously reviewed at another journal that is not operating a transparent peer review scheme. This document only contains reviewer comments and rebuttal letters for versions considered at *Nature Communications*.

Reviewers' Comments:

Reviewer #1:
Remarks to the Author:
Thank you for addressing all of the reviews of your first submission. The new version of the paper is greatly improved and I appreciate the work you did to get it to this point.

Reviewer #2:
Remarks to the Author:
The authors have addressed my comments and questions adequately. The paper has improved significantly compared to the first version. Well done!

Reviewer #3:
Remarks to the Author:
I have reviewed the revised version of the manuscript alongside my original review. My concerns have been adequately addressed and I believe the manuscript is suitable for publication in Nature Communications. It is a nice piece of work that will be welcomed by the RNA synthetic biology community.
